# Supplementary figures and images for: HDAC5 Inhibitors as a Potential Treatment in Breast Cancer Affecting Very Young Women
Source: Cancers (Basel). 2020 Feb 10;12(2):412. doi: 10.3390/cancers12020412 (PMC7072585; doi:10.3390/cancers12020412)

Figure S1

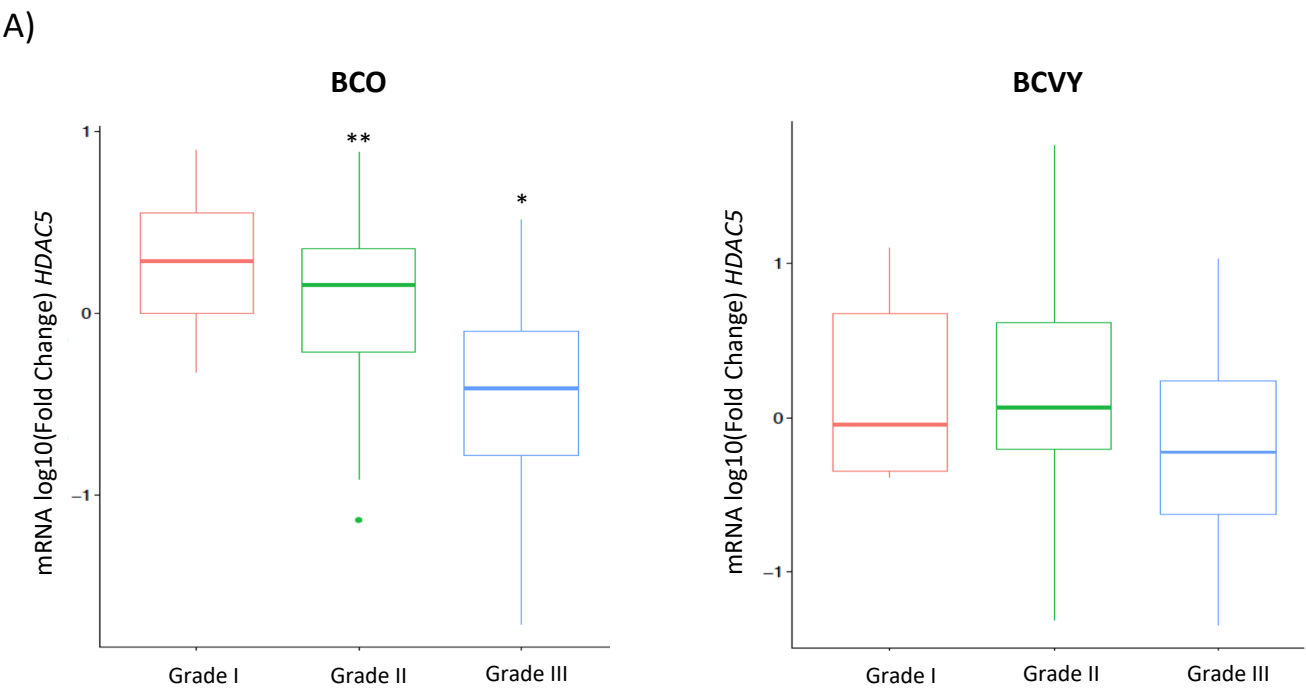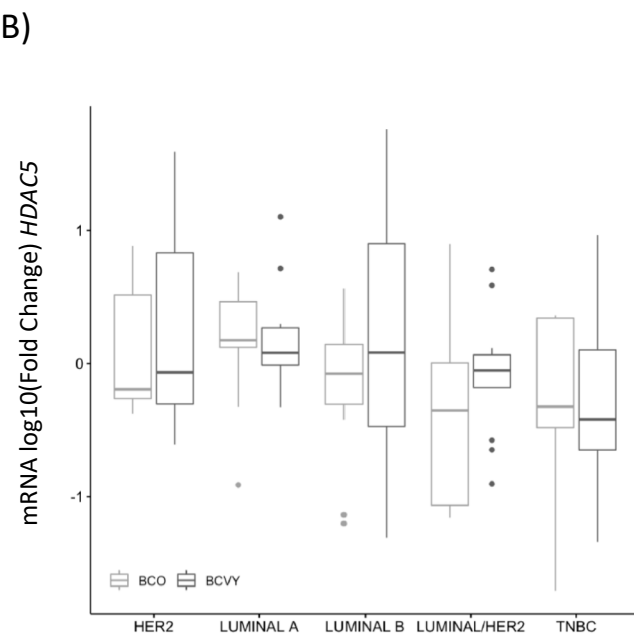

Supplement: Supplementary file 1 [file cancers-12-00412-s001.pdf]
